# Progenitor Cells from Cartilage: Grade Specific Differences in Stem Cell Marker Expression

**DOI:** 10.3390/ijms18081759

**Published:** 2017-08-12

**Authors:** Marija Mazor, Annabelle Cesaro, Mazen Ali, Thomas M. Best, Eric Lespessaille, Hechmi Toumi

**Affiliations:** 1 Department of Sciences, University of Orleans, I3MTO, EA 4708, Orleans F-45032, France; mazor.marica@gmail.com (M.M.); annabelle.cesaro@univ-orleans.fr (A.C.); eric.lespessailles@chr-orleans.fr (E.L.); 2 Service chirurgie orthopédique et traumatologique Centre Hospitalier Régional d’Orléans, La Source 45000, France; mazen.ali@chr-orleans.fr; 3 UHealth Sports Medicine Institute, Department of Orthopedics, Division of Sports Medicine, U of Miami, Coral Gables, FL 33146, USA; txb440@med.miami.edu; 4 EA4708/I3MTO, Service de Rhumatologie, Centre Hospitalier Régional d’Orléans, La Source 45000, France

**Keywords:** mesenchymal stem cell-like progenitors, osteoarthritic, cartilage

## Abstract

Recent research has confirmed the presence of Mesenchymal stem cell (MSC)-like progenitors (MPC) in both normal and osteoarthritic cartilage. However, there is only limited information concerning how MPC markers are expressed with osteoarthritis (OA) progression. The purpose of this study was to compare the prevalence of various MPC markers in different OA grades. Human osteoarthritic tibial plateaus were obtained from ten patients undergoing total knee replacement. Each sample had been classified into a mild or severe group according to OARSI scoring. Tissue was taken from each specimen and mRNA expression levels of CD105, CD166, Notch 1, Sox9, Acan and Col II A1 were measured at day 0 and day 14 (2 weeks in vitro). Furthermore, MSC markers: Nucleostemin, CD90, CD73, CD166, CD105 and Notch 1 were studied by immunofluorescence. mRNA levels of MSC markers did not differ between mild and severe OA at day 0. At day 14, protein analysis showed that proliferated cells from both sources expressed all 6 MSC markers. Only cells from the mild OA subjects resulted in a significant increase of mRNA CD105 and CD166 after in vitro expansion. Moreover, cells from the mild OA subjects showed significantly higher levels of CD105, Sox9 and Acan compared with those from severe OA specimens. Results confirmed the presence of MSC markers in mild and severe OA tissue at both mRNA and protein levels. We found significant differences between cells obtained from mild compared to severe OA specimens suggests that mild OA derived cells may have a greater MSC potential.

## 1. Introduction

Osteoarthritis (OA), the most common degenerative joint disease, is a consequence of mechanical and biological events that destabilize tissue homeostasis and cause destruction of articular joints [1]. While various options are available for the clinician to attempt repair of the damaged joint surface, none can reliably restore natural articular cartilage integrity. Current treatment techniques include microfracture, chondrocyte implantation, and osteochondral grafting [1,2]. However, the regenerated cells fail to organize a healthy layered structure and the resulting fibrocartilage demonstrates a variable architecture that is biochemically and biomechanically different from healthy articular cartilage [2,3]. Consequently, there has been a longstanding interest in improving articular cartilage repair through the implantation of chondrocyte progenitor cells into symptomatic chondral defects.

Protocols based on the delivery of mesenchymal stem cells (MSC) are currently showing the most encouraging results for the treatment of articular cartilage lesions, perhaps due to their self-renewal properties and capacity to form specialized tissue [4,5,6]. These cells have been found in various adult tissues including bone marrow [5], adipose tissue [7] and synovium [8]. However, the limitation in using MSC from these sources is that stable chondrogenic differentiation has not yet been achieved due to the hypertrophic potential, which leads the cells through the enchondral ossification pathway toward bone formation [9,10]. This had been demonstrated by in vitro chondrogenesis of MSC which resulted in higher expression of hypertrophic markers (collagen type X and MMP13) and production of fibrocartilaginous rather than healthy hyaline cartilage tissue [11,12,13]. As a result, articular cartilage repair and regeneration continue to be largely intractable. It has been shown that MSC can be found in nearly all tissues, have migratory abilities, and act as a primary matrix for tissue regeneration during inflammation and tissue injury [14,15,16]. Hence, many researchers have focused on exploring the presence of MSC-like progenitors (MPC) in human cartilage tissue itself [17,18,19,20]. Such cells may have a high potential in cartilage repair strategies as they could possess the developmental repertoire of the native tissue and positively impact the morphogenesis of the repair tissue. Many protocols have investigated MPC potential from both OA cartilage and healthy cartilage tissues [17,18,19,20,21,22,23]. Criteria applied to define MPC presence in cartilage tissue include: expression of MSC related markers (i.e., CD105, CD166, Stro 1, CD106, CD90, CD73 etc.), clonogenicity and multilineage differentiation ability [24,25].

Phenotypic analysis of cells isolated from healthy cartilage reported the presence of certain sub–populations express MSC related markers, individually or in combination, including: CD105^+^CD166^+^ [17,22,26], Stro-1^+^ [27], Notch-1^+^ [19,20], CD166^+^CD90^+^ [28], CD106^+^Stro-1^+^Notch-1^+^ [20,21]. Interestingly, several authors observed a higher expression of these markers in osteoarthritic compared with normal cartilage [20,22,26,29]. Two studies reported that CD105^+^CD166^+^ positive cells were 2% (Pretzel et al.) and 3% (Alsalameh, S. et al.) higher in osteoarthritic than in normal human cartilage, respectively [22,26]. Similar results have been observed by Hiraoka K et al., (2006) for the Notch-1^+^ MSC marker. Unlike normal cartilage, a high portion of OA cartilage cells stain positive for Notch-1 [20]. According to Fickert and coworkers, 2–12% of cells isolated from OA cartilage corresponded to cells with MPC characteristic (CD9^+^CD90^+^CD166^+^) [29]. The increase of MPC in OA cartilage may be a natural response to damage in order to replace dead chondrocytes and to maintain new matrix deposition. In vitro studies of cartilage explants have showed emergence of cartilage progenitors at the injured site with the capacity to differentiate to mature chondrocytes and depose de novo synthetized matrix [30,31]. In addition, in situ studies have demonstrated that cell clusters are localized near and within the fissures and clefts of OA cartilage with most cells expressing Notch-1^+^Stro-1^+^CD106^+^ MSC markers [21,32,33]. Taken together, several researchers have confirmed the potential interest of progenitors derived from cartilage tissue to assist with cartilage repair. The purpose of this study was to answer to the question whether MSC-like progenitor cell potential varies with OA progression. To that end, we quantified MSC markers in tissue and proliferated cells (after two weeks of proliferation) isolated from articular cartilage of mild and severe OA specimens.

## 2. Results

**Figure 1:**
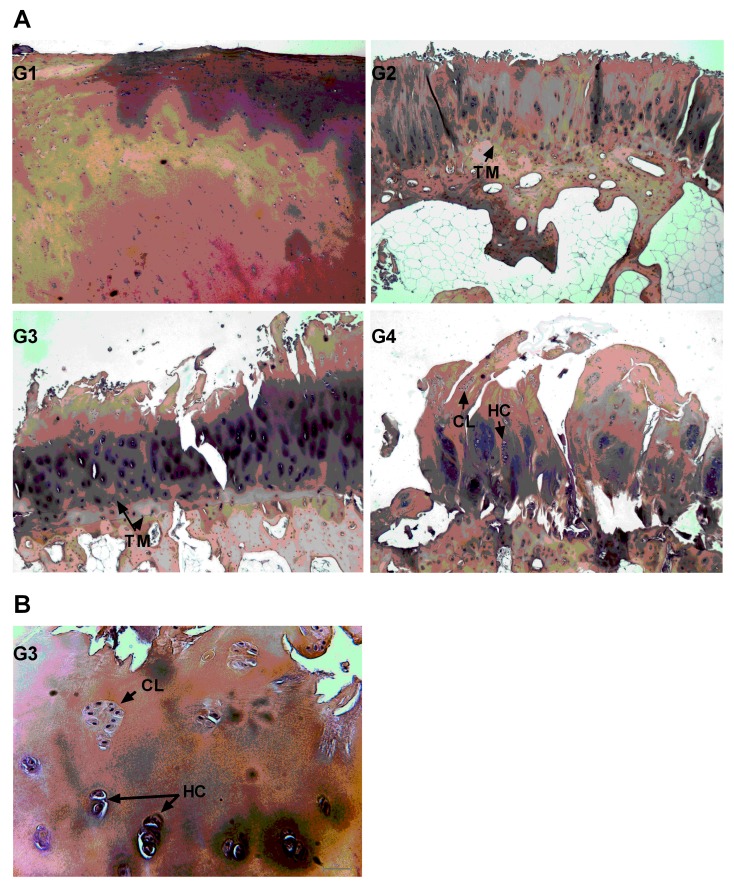
OARSI scoring assessment of specimen. (**A**) HES stained sections of cartilage at different grades of degradation-Grade 1/G1: superficial fibrillation and cells death; Grade 2/G2: matrix depletion in superficial and middle zone and disorientation of chondrocytes columns (black arrows); Grade 3: matrix vertical fissures into mid zone, formation of the chondrocytes clusters and hypertrophic chondrocytes (black arrows); Grade 4: loss of superficial and mid zone of cartilage, increase in the number of clusters and hypertrophic chondrocytes (black arrows) (4×); (**B**) Chondrocytes clusters and hypertrophic chondrocytes (20×).

Pathological changes of 10 osteoarthritic tibial plateaus were evaluated based on the OARSI scoring assessment [34] (Figure 1). Samples were divided into two groups: mild OA (grade 1 & 2) and severe OA (grade 3 & 4). mRNA expression levels of MSC and chondrocytes markers were firstly measured in the tissue (D0) then in cells after two weeks of proliferation (D14). The expression of MSC markers in proliferated cells was further confirmed at the protein level. The main findings of our study are presented below.

**Figure 2:**
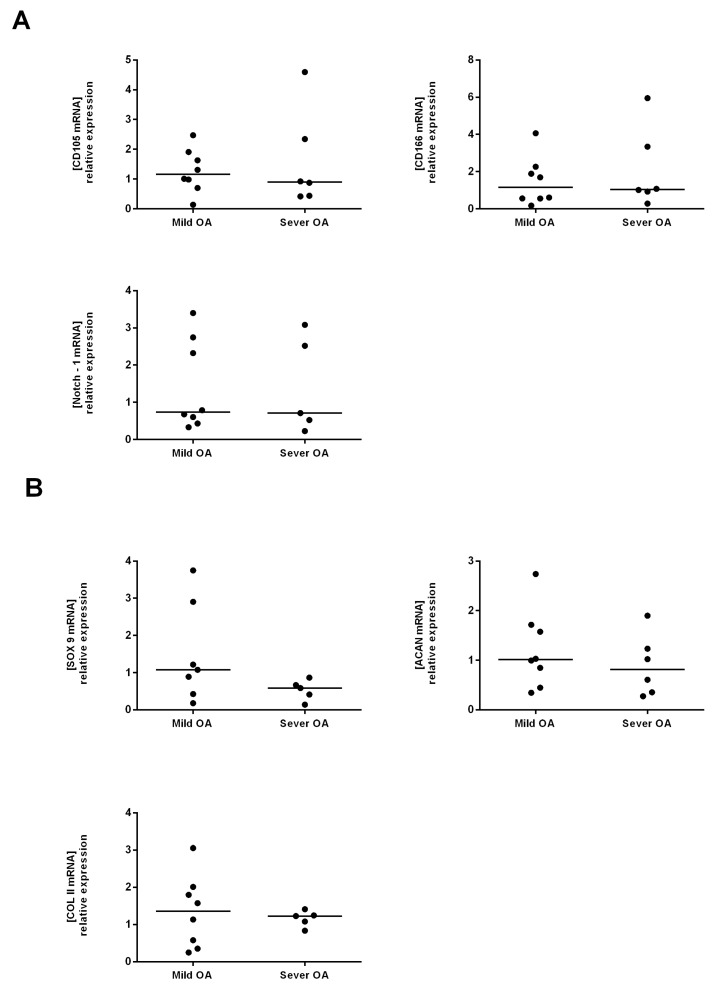
Comparative analysis of MSC and Chondrocyte markers in the tissue (D0) isolated from mild and severe OA regions. (**A**) The mRNA expression levels of CD105, CD166 and Notch 1 (MSC markers) in mild versus severe OA at D0; (**B**) The mRNA expression levels of Sox9, Acan and Col II (chondrocyte markers) in mild versus severe OA at D0. Mild OA is used as the control expression to normalized results.

Tissue isolated from the mild and severe OA regions did not significantly differ in the mRNA expression of MSC markers; CD105, CD166 and Notch 1 (Figure 2A). Similar results were observed for the chondrocyte markers: Sox9, Col II A1 and Acan (Figure 2B)*.*

**Figure 3:**
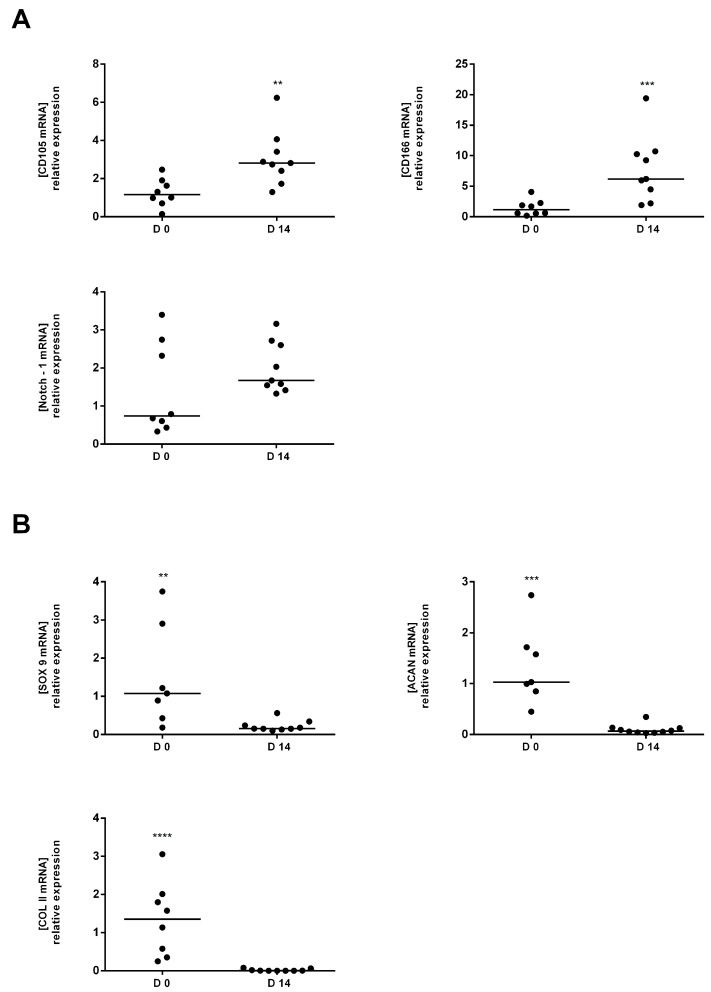
Comparative analysis of MSC and Chondrocyte markers between tissue (D0) and proliferated cells (D14) in Mild OA. (**A**) The mRNA expression levels of CD105, CD166 and Notch 1 (MSC markers) at D14 versus D0; (**B**) The mRNA expression levels of Sox9, Acan and Col II (chondrocytes markers) at D14 versus D0. D0 is used as the control expression to normalise results. ** *p* < 0.01, *** *p* < 0.001, **** *p* < 0.0001, Student’s *t*-test (*n* = 8 in Mild OA, *n* = 6 in severe OA).

Cells obtained from mild OA cartilage showed significantly higher mRNA levels of CD105 (*p* = 0.0037) and CD166 (*p* = 0.0010) after in vitro culture (D14) compared to harvested day (D0). The rate of increase was 2 and 5 fold for CD105 and CD166 respectively (Figure 3A). Yet, Notch 1 did not reveal significant increases between D0 and D14 (Figure 3A). Furthermore, we compared the expression levels of CD105 and CD166 (D14) to those resulted from MSC commercial cell line (MSC Human, Product sub category: Primary Cells, Target species: Human, Tissue type: Bone marrow, Shipment info: Liquid Nitrogen, Source: HemaCare Corp, Paris, France).

**Figure 4:**
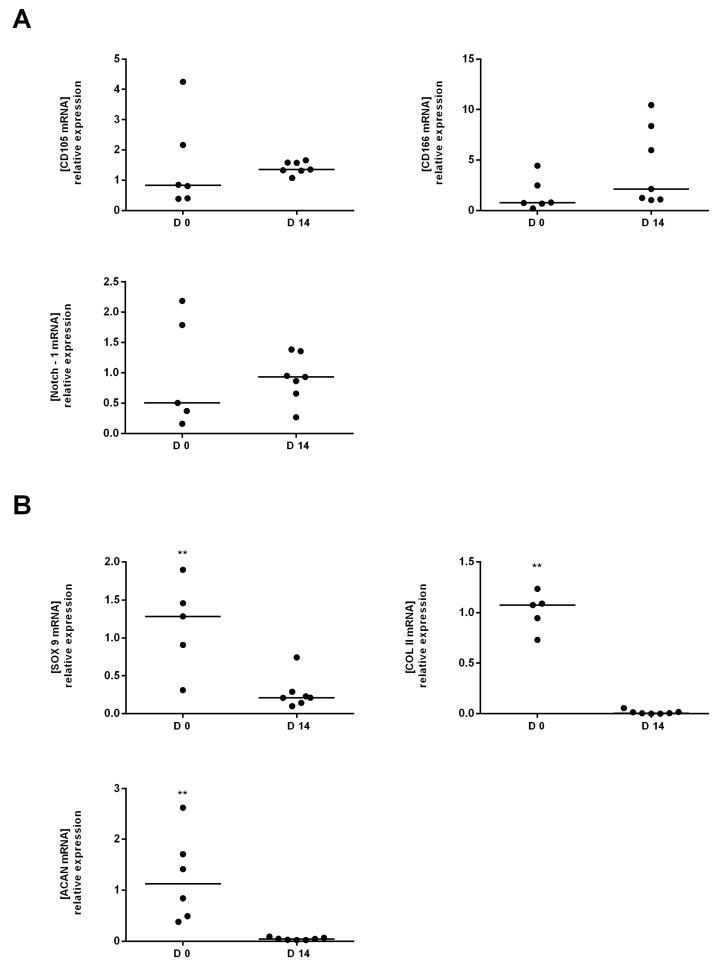
Comparative analysis of MSC and Chondrocytes markers between tissue (D0) and proliferated cells (D14) in severe OA. (**A**) The mRNA expression levels of CD105, CD166 and Notch 1 (MSC markers) at D14 versus D0; (**B**) The mRNA expression levels of Sox9, Acan and Col II (chondrocytes markers) at D14 versus D0. D0 is used as the control expression to normalized results. ** *p* < 0.01, Student’s *t*-test (*n* = 8 in Mild OA, *n* = 6 in severe OA).

The scores showed that the MSC cell line (MSC commercial cell line) didn’t express a significant difference for CD105 and CD166 compared to our cells (cells obtained from total knee replacement surgery). The mRNA expression of chondrocyte markers: Sox 9, Acan and Col II A1 significantly decreased at D14 compared to D0. The increase was 7, 14 and 75 times for Sox9, Acan and Col II A1, respectively (Figure 3B). Results were mirrored for cells obtained from severe OA cells (Figure 4B). Conversely, mRNA expression levels of the CD105 and CD166 between D0 and D14 were not significantly increased for the cells isolated from severe OA cartilage (Figure 4A). In addition, mild OA derived cells showed a higher proliferation rate after 2 weeks in vitro compared to the severe OA derived cells. When day 0 (D0) was compared to day 14 (D14) cell proliferation rate were 7.97 ± 6.198 and 4.97 ± 2.39 for mild OA and severe OA cartilage, respectively.

**Figure 5:**
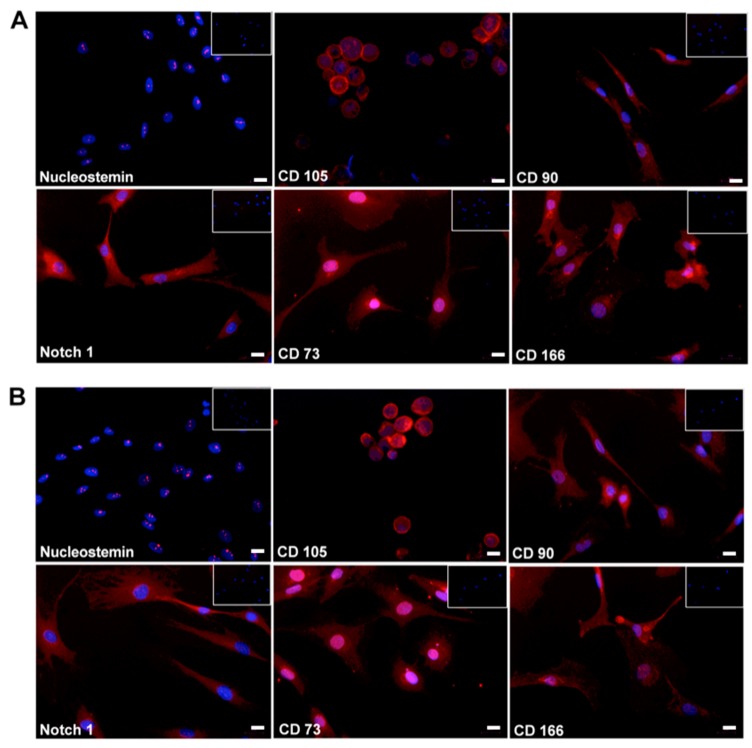
Protein expression of MSC markers in Mild and severe OA-derived cells at D14 (scale bar 20 µm). Immunofluorescence of Nucleostemin, CD105, CD166, CD73, CD90 and Notch 1 in proliferated cells (D14) isolated from areas of Mild OA (**A**) and severe OA (**B**). Markers of interest-RE staining (Red), contra staining DAPI (blue), and Isotype control + DAPI-left corner of each image.

**Figure 6:**
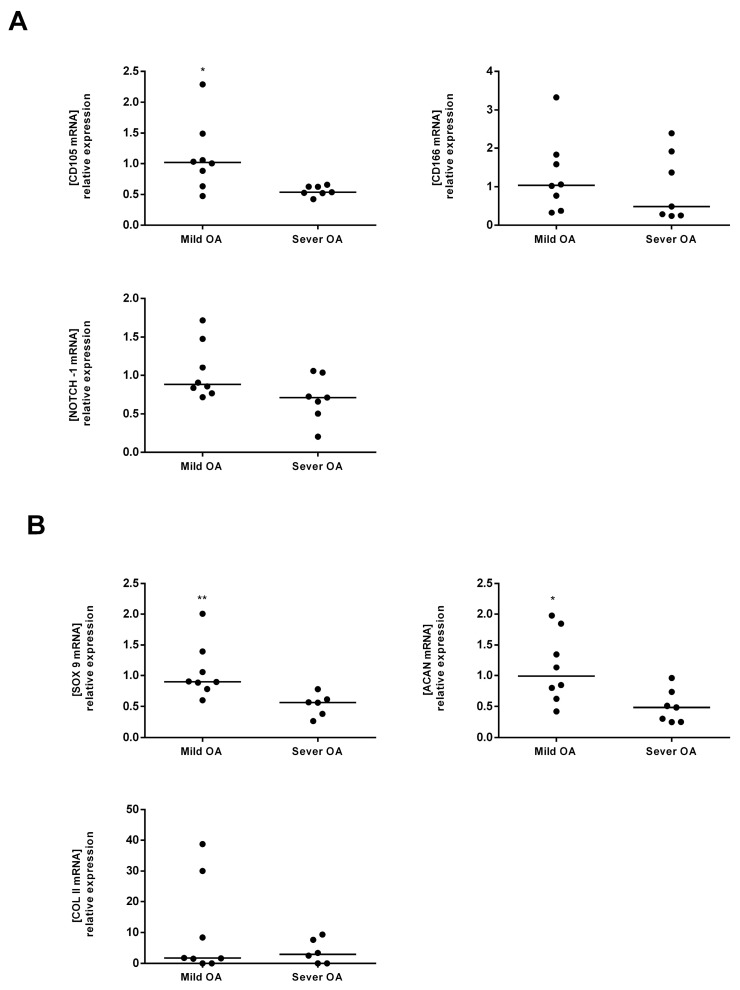
Comparative analysis of MSC and Chondrocyte markers in proliferated cells (D14) isolated from mild and severe OA regions. (**A**) The mRNA expression levels of CD105, CD166 and Notch 1 (MSC markers) in mild versus severe OA at D14; (**B**) The mRNA expression levels of Sox9, Acan and Col II (chondrocytes markers) in mild versus severe OA at D148. Mild OA is used as the control expression to normalized results. * *p* < 0.05, ** *p* < 0.01, Student’s *t*-test (*n* = 8 in Mild OA, *n* = 6 in severe OA).

Both proliferated cells (D14) derived from mild (Figure 5A) and from severe OA (Figure 5B) showed positive staining for 6 MSC markers; CD90, CD73, CD166, Nucleostemin, CD105 and Notch-1. At D14, mild-OA derived cells expressed a significantly higher level of CD105 (2 fold) compared to severe-OA derived cells (Figure 6A). Yet, no significant difference has been found for the CD166 and Notch 1 markers (Figure 6A). Sox9 and Acan showed a significantly higher expression (1.5 fold and 2 folds respectively) in Mild OA compared to severe OA cells (Figure 6B)*.*

## 3. Discussion

The aim of the present study was to compare the extent of MSC-like progenitors, depending on the degree of morphological OA-related damage to achieve this, OA cartilage-harvested tissue and proliferated cells were examined for the expression of general MSC markers of mild OA compared to severe OA specimens. Our study demonstrated that only proliferated cells obtained from mild OA tibial plateau specimens demonstrated increased mRNA levels of CD105 and CD166 after 2 weeks of in vitro expansion. Yet, phenotype analysis at D14 demonstrated that cells from both mild and severe OA specimens expressed the same MSC specific proteins: CD73, CD90, CD105, CD166, Nucleostemin and Notch 1. Nevertheless, analysis at D14 compared with Day 0 showed that mild OA derived cells expressed higher mRNA levels of CD105 and Sox9 compared to severe OA derived cells. This finding suggests to us that mild OA derived cells may have a greater MSC potential than severe ones. The clinical significance of this finding remains uncertain but may have implications for the timing of cell-based therapies in the treatment of individuals with knee OA.

Herein, both mild and severe OA cartilage at D0 expressed increased CD105, CD166 and Notch 1 mRNA expression. Previous studies confirmed the presence of these three markers in normal and/or OA human articular cartilage [17,22,26]. Alsalameh et al., 2004 reported that the prevalence of CD105^+^CD166^+^ positive cells in OA was around 3% higher in cells obtained from OA cartilage compared to normal [26]. More recently, Hiraoka et al., 2006 demonstrated that Notch 1^+^ cells were increased in OA cartilage compared to healthy. Notch 1^+^ cells were mostly present in cell clusters of severely damaged OA tissue [20]. Our study showed no significant difference in mRNA markers between mild and severe OA tissue at D0. Of note, we compared tissues from severe to mild OA and not severe to healthy as previously reported [20,22,26]. Furthermore, we compared mRNA levels of MSC markers and Alsalameh et al., 2004 compared protein levels of primary cell cultures while Hiraoka et al., 2006 examined in situ changes.

Following in vitro proliferation, only mild OA derived cells showed a significant increase of CD105 and CD166 mRNA. It has been reported that cell adherence and ability to grow on plastic is one of the MSC characteristics [24,35]. In our protocol, proliferation assay of isolated cells was performed on plastic without previous coating and a higher proliferation capacity was found for mild OA derived cells compared to severe OA. Proliferation assay and MSC gene expression results suggest a higher MSC prevalence in mild OA derived cells compared to severe OA.

Our immunofloresence results showed that cells isolated from both mild and severe OA cartilage stained positively for CD73, CD90, CD105, CD166 and Notch 1, which could indicate the presence of MSC progenitors. This is in line with a previous report by Alsalameh et al., 2004 demonstrating positive staining of these markers in OA and normal cartilage cells including CD105^+^CD166^+^. Another study performed on proliferated cells obtained from normal human cartilage stained positively for Notch 1 receptor, ligands δ 1, Jagged 1, and MSC markersCD90 and Stro-1 [17]. The Notch 1 signaling pathway regulates stem cell proliferation, self-renewal and upregulation of mesenchymal phenotypes [36]. It has been shown that Nucleostemin is a marker for stem-cell proliferation [37,38,39,40]. Therefore, we additionally investigated nucleostemin expression by immunofluorescence and showed its presence in all expanded cells. Our study for the first time demonstrated positive Nucleostemin staining in OA proliferated cells.

In order to quantify any differences between mild and severe at day 14, we performed mRNA analysis for MSC markers. Results showed higher levels of CD105 and Sox9 in mild compared to severe OA. Sox9 is an earlier chondrogenesis marker and known to promote the MSC condensation that undergo morphological changes when associated with early chondrogenic differentiation [41,42,43,44]. Thus, it could be suggested that the high incidence of this transcription factor in expanded mild OA-derived cells may indicate that these cells could maintain great chondrogenetic potential even after monolayer expansion. Moreover, the high level of Sox9 was associated with high expression of aggreacan in mild OA-derived cells. This observation supports previous findings and suggests that Sox9 enhances aggrecan promoter activity [45]. Pretzel and co-workers demonstrated that the prevalence of CD105^+^CD166^+^ positive cells were 15% and 17% for normal and OA cartilage cells respectively [22]. These authors compared normal cartilage tissue obtained from donors with no known history of joint disease to tissue obtained from patients who had undergone total joint replacement surgery [22,26]. In contrast, our study compared different degrees of morphological OA-related damaged tissues, which may provide insight as to when MPC cells are most abundant in relation to OA progression similar to a previous study [46]. Differing with our study, sample grading was done according to Outerbridge’s classification and not confirmed histologically. Cells isolated from OA cartilage at the third passage expressed 50–70% of MSC markers CD9^+^CD105^+^CD73^+^ irrespective of the stage of the original cartilage degradation [46]. The cells were collected at third passage whereas ours were collected at second passage and this could effect cell phenotypic expression [46]. Effectively, previous reports have shown that longer proliferation period induces dedifferentiation of mature chondrocytes and express putative markers which may overlap with markers expressed by potentially present progenitors [47,48,49,50].

## 4. Materials and Methods

### 4.1. Cartilage Preparation

Human osteoarthritic (OA) tibia plateaus were obtained from the knee joints of 10 patients (58 to 85 years; mean 70 years) diagnosed with various grades of OA undergoing total knee joint arthroplasty. Patients with systemic inflammatory diseases such as rheumatoid arthritis or spondyloarthropathies were excluded. The protocol was approved by the CPP (Comité de Protection des Personnes Nord Ouest IV, Protocole RT-07, biological collection: CB.2012.RT.07, 23rd February 2012) and patients gave their consent for the use of samples for research.

**Figure 7:**
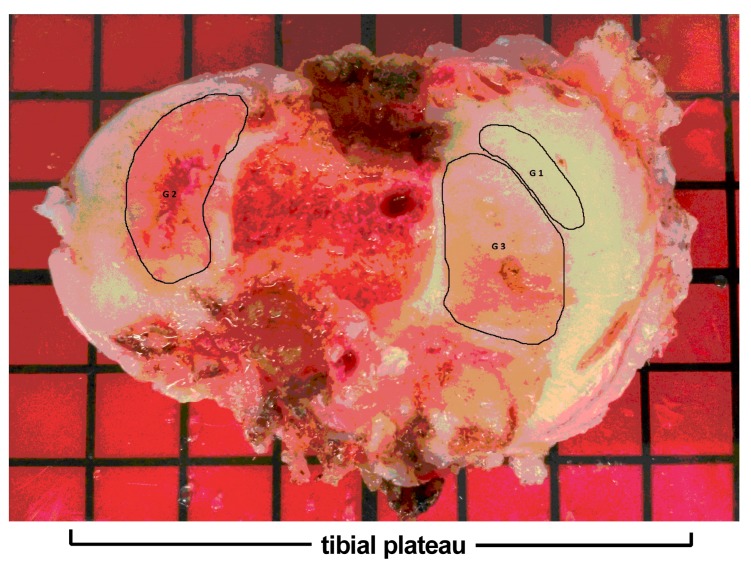
Representative OA tibial plateau obtained after a total knee joint replacement surgery. The Outerbridge’s classification was used to determine the grade of the interesting area indicated by black line (Grade 1/G1: minimal cartilage degradation; Grade 2/G2: partial-thickness defect with fissures on the surface; Grade 3/G3: fissures to the level of subchondral bone.

OA tibial plateaus were macroscopically graded according to the Outerbridge classification (Grade 0: intact cartilage; Grade 1: minimal fibrillation and softening; Grade 2: partial-thickness defect with fissures on the surface; Grade 3: fissuring to the level of subchondral bone; Grade 4: total loss of cartilage and exposure of subchondral bone (Figure 7). Samples from areas with different grades were harvested and used for the experiments.

### 4.2. Histological Assessment of OA Grafts

Grafts of 8 mm in diameter were isolated from OA tibial plateau and fixed in paraformaldehyde 4% (Sigma-Aldrich, Lyon, France), decalcified in EDTA 15% (Sigma-Aldrich, Lyon, France) for 3 weeks and embedded in paraffin. Sections (5 µm) were stained with HES (Merck Millipore, Fontenay sous Bois, France) and scored by two blinded observers according to the OARSI scales (grade 0: intact cartilage surface, normal matrix morphology and intact cells orientation; Grade 1: superficial fibrillation and cells death and hypertrophy; Grade 2: matrix depletion in superficial and middle zone and disorientation of chondrocytes columns; Grade 3: matrix vertical fissures into mid zone and formation of the chondrocytes clusters; Grade 4: loss of superficial and mid zone of cartilage, grade 5 and 6: no cartilage left and bone deformation is present) [34].

### 4.3. Isolation and Culture of Cells from Cartilage Samples

For cell isolation, cartilage slides form each grade were harvested with scalpel and washed twice in PBS supplemented with penicillin/streptomycin (PS) 1% (Gibco™, Courtaboeuf, France). Slides were then incubated for 4 h at 37 °C in serum-free DMEM/F12 (Gibco™, Courtaboeuf, France) containing collagenase B 0.2% (Roche, Mannheim, Germany). Supernatant were then collected and centrifuged under 2000 rpm to pellet cells. Cells were washed once in PBS/1% PS, counted and seeded in DMEM/F12 supplemented with FBS 10%, PS 1% and Fungizon 0.1% (Gibco™, Courtaboeuf, France). A new enzymatic digestion was performed on the remaining undigested tissue. Cells were cultured up to 2 weeks in DMEM/F12 supplemented with FBS 10%, PS 1% and Fungizon 0.1%. At 80% confluence, cells were dissociated by trypsin treatment (Gibco™, Courtaboeuf, France) and cultured in order to obtain sufficient numbers of cells. Media was changed three times a week. Cell proliferation rate was calculated dividing the number of cells after two weeks of proliferation by the number of cells initially seeded.

### 4.4. mRNA Expression/Quantitative Real-Time PCR

Total RNA was extracted from both cartilage tissue (D0) and 2 week expanded (D14) cells with the RNeasy mini kit according to the manufacturer’s instructions (Qiagen, Paris, France). The tissue homogenization was pre-performed in liquid nitrogen with the Handheld Homogenizer (ProScientific, Oxford, CT, USA). CDNA obtained with the Quanti Tect Reverse Transcription kit according to the manufacturer’s instructions (Qiagen, Paris, France). QPCR amplification was carried out with the CFX96 Real Time System (Bio-Rad, Marnes-la-Coquette, France). Cells and tissue were tested on the expression of the following primers: CD105 (Cat. No. QT00013335, Qiagen, Paris, France), CD166 (Cat. No. Q100026824, Qiagen, Paris, France), Notch 1 (Cat. No. QT00001365, Qiagen, Paris, France), ACAN (Cat. No. QT00001365, Qiagen, Paris, France), Col II A1 (Cat. No. QT00049518, Qiagen, Paris, France). Sox9 (5′GCTCTGGAGACTTCTGAACGA3′, 5′GGGAGATGTGCGTCTGCT3′) primers were synthesized by Invitrogen™ (ThermoFisher Scientific, Courtaboeuf, France). Expression of the housekeeping gene GAPDH was used to normalize gene expression in the analysis (Cat. No. QT01192646, Qiagen, Paris, France). The 2-ΔΔCt method was used for relative gene expression analysis.

### 4.5. Immunofluorescence

Cells were expanded for 2 weeks in DMEM/F12 supplemented with FBS 10%, PS 1% and Fungizon 0.1% (Gibco™, Courtaboeuf, France) and then subjected to immunofluorescence for antibodies to anti-goat Notch 1 (1:50; sc-9170, Santa Cruz Biotechecnology^®^, Inc., Dallas, TX, USA), anti-rabbit CD90 (1:50; sc-9163, Santa Cruz Biotechecnology^®^, Inc.), anti-mouse CD166 (1:50; sc-74557, Santa Cruz Biotechecnology^®^, Inc., Santa Cruz, CA, USA), anti-rabbit nucleostemin (1:100; ab 70346, Abcam^®^, Paris, France), anti-rabbit CD73 (1:50; ab175396, Abcam^®^, Paris, France) and R-phycoerythrin-conjugated anti-CD105 (clone SN6; IgG1; Ancell Corporation, Bayport, MN, USA). The CD105 staining was performed on the cells suspension, while the other antibodies were tested on adherent cells. Before staining, adherent cells were fixed (4% paraformaldehyde/20 min/RT, 2% paraformaldehyde/10 min/RT, or 100% methanol/5 min/−20 °C), permeabilized (PBS-Triton 0.1%-Triton 100x; Acros organics, Geel, Belgium) and non-specific sites were blocked with 1% bovine serum albumin for 1 h. Cells were then incubated with primary antibodies overnight at 4 °C in a humid chamber. After the primary antibody, cells were incubated for 1 h at room temperature with the 549-conjugated anti-rabbit, 549-conjugated anti-mouse or 549-conjugated anti-goat antibodies (1:1500; Rochland, Limerick, Ireland). The control slides were treated only with the secondary antibody and served as a negative control. Cell suspensions were subjected to the R-phycoerythrin-conjugated anti-CD105, pretreated with trypsin, washed twice with PBS1x and non-specific site were blocked for 45 min in 1% bovine serum albumin. The staining was performed at room temperature for 1 h. After staining, cells were fixed with 2% paraformaldehyde for 10 min. Cell nuclei of both the adherent and cell in suspension was stained with DAPI (4’,6-diamidino-2-phenylindole) and cells were mounted in immunostaining mounting solution. Fluorescence-labeled cells were visualized under the ×40 magnification with a BA400 microscope (Motic^®^, Wetzlar, Germany). Cells incubated only with the-secondary antibodies showed no staining.

### 4.6. Statistical Analysis

Data are reported as the mean ± standard error of the mean. The results were analyzed with non-parametric Mann-Whitney test. The critical *p-*value for statistical significance was *p* = 0.05.

## 5. Conclusions

Our study confirmed the presence of MSC-like progenitors in the tibial plateau of both mild and severe OA specimens. Interestingly, mild OA derived cells demonstrated significantly increased mRNA levels of MSC markers between D0 and D14 while severe OA derived cells did not, suggesting potentially important differences may exist for chondrogenic potential depending on the severity of OA. To that end, mild OA derived cells may have a greater MSC potential than similar severe OA cells. The clinical implications of this finding remain unknown but provide some preliminary insights for timing of stem cell-based therapies and their treatment of OA.
